# Assessment of the learning environment among medical students at Tabuk University, Saudi Arabia: a cross-sectional study

**DOI:** 10.3389/fmed.2026.1816941

**Published:** 2026-05-29

**Authors:** Albaraa A. Altowijri

**Affiliations:** Department of Surgery, Faculty of Medicine, University of Tabuk, Tabuk, Saudi Arabia

**Keywords:** academic burnout, cross-sectional study, learning environment, medical education, Saudi Arabia, student satisfaction

## Abstract

**Background:**

The learning environment (LE) is integral to medical students’ academic performance, emotional well-being, and professional development. Students’ perceptions of their learning environment provide valuable insights for identifying institutional strengths and weaknesses.

**Purpose:**

This study aimed to assess the perceived learning environment among medical students at Tabuk University and to examine differences across academic years and between male and female students.

**Methods:**

A quantitative cross-sectional study was conducted in December 2023 among medical students at Tabuk University. A total of 304 students participated. Data were collected using a 53-item self-administered questionnaire adapted from established instruments, including the Dundee Ready Education Environment Measure (DREEM) and the Medical School Learning Environment Survey (MSLES). The questionnaire covered domains related to teaching quality, academic support, student well-being, social integration, and curriculum relevance, and its content validity was established before use. Responses were recorded on a 5-point Likert scale. Data analysis included descriptive statistics, independent-samples *t*-tests, one-way ANOVA, and Pearson correlation analysis.

**Results:**

The response rate was 31.7% (304/960). Participants represented all academic years and were fairly balanced by gender. The overall mean LE satisfaction score was 3.52 (SD = 0.81), indicating a moderately positive perception. A clear U-shaped pattern was observed across the six academic years, with the lowest satisfaction in the second year and the highest in the sixth year. Female students reported significantly higher satisfaction than male students (*p* < 0.001). Teacher knowledge and peer support emerged as key strengths, whereas student fatigue and perceptions of academic dishonesty were identified as major concerns.

**Conclusion:**

The learning environment at Tabuk University was perceived as moderately positive, as reflected by an overall mean score above the neutral midpoint (3.0) of the questionnaire. However, notable challenges remain, particularly during the preclinical years. Efforts to strengthen student well-being, academic support, and curriculum design may further improve the learning environment.

## Introduction

The learning environment (LE) plays a central role in medical education. It influences not only academic achievement but also students’ well-being, professional identity formation, and overall educational experience (1, 2). A supportive and positive learning environment can enhance motivation, engagement, and academic success, whereas a negative environment may contribute to stress, anxiety, and burnout (3, 4). Several instruments have been developed to assess the LE, including the Dundee Ready Education Environment Measure (DREEM) and the Medical School Learning Environment Survey (MSLES) (5, 6). These tools help identify important aspects of the educational climate, such as teaching quality, academic support, and student-teacher relationships, and are useful for identifying areas that require improvement (7). Medical education has changed considerably in recent years, particularly in Saudi Arabia and other Gulf Cooperation Council (GCC) countries. Reforms such as competency-based medical education (CBME), problem-based learning (PBL), and early clinical exposure have reshaped medical curricula (8–10). In addition, hybrid and technology-enhanced learning following the COVID-19 pandemic has created both new opportunities and new challenges (11). These developments highlight the importance of continuously evaluating how students perceive their current learning environment. Student well-being has also become a major concern in medical education. International evidence shows that medical students commonly experience high levels of stress, anxiety, and burnout (12, 13), and the environment in which they learn is an important contributing factor. Therefore, ongoing assessment of the LE is essential for improving both academic outcomes and student well-being (14).

In Saudi Arabia, studies examining medical students’ perceptions of the learning environment have produced inconsistent findings and have often been limited to specific departments or academic years (15, 16). There is still limited institutional evidence describing how these perceptions change across the full 6 years of medical training. This gap is particularly important for developing medical schools such as Tabuk University, where curriculum development requires continuous feedback and regular evaluation. Identifying the strengths and weaknesses of the learning environment can guide targeted improvements in medical education. Therefore, this study aimed to assess the perceived learning environment among medical students at Tabuk University and to compare differences by academic year and gender in order to identify areas of the educational process that may require further improvement.

## Methods

### Study design and setting

This quantitative cross-sectional study evaluated medical students’ perceptions of the learning environment. It was conducted in December 2023 at the College of Medicine, University of Tabuk, Saudi Arabia. The medical program at the University of Tabuk follows a 6-year MBBS curriculum divided into two main phases:

- Preclinical phase (Years 1–3): focuses on the foundations of medical sciences through integrated modules that include subjects such as anatomy, physiology, and biochemistry, together with early clinical exposure.
- Clinical phase (Years 4–6): involves hospital-based rotations in major specialties, including internal medicine, surgery, pediatrics, and obstetrics and gynecology. This phase is designed to provide a structured transition from theory to practice, with progressively increasing clinical responsibility.

The target population comprised all undergraduate medical students in Years 1–6 who were registered during the 2023–2024 academic year. A census sampling approach was used, in which all eligible students were invited to participate. A total of 304 students completed the questionnaire and were included in the final analysis.

### Study population and sampling

The target population comprised all undergraduate medical students in the first through sixth academic years at the College of Medicine, University of Tabuk, during the 2023–2024 academic year.

A full-enumeration (census) sampling strategy was adopted, and all eligible students were invited to participate. The final sample consisted of 304 respondents who completed the survey, representing a substantial cross-section of the student body. Demographic characteristics included academic year (first through sixth year), sex according to institutional records, and self-reported age.

The inclusion criteria were as follows:

- Medical students (Years 1–6, 2023–2024 academic year).
- Students who agreed to complete the electronic questionnaire independently.

The exclusion criteria were as follows:

- Incomplete or partially completed questionnaires (excluded when cleaning the data).

### Data collection instrument and variables

Data were collected using a 53-item self-administered questionnaire developed by the research team and informed by well-established instruments, namely the Dundee Ready Education Environment Measure (DREEM) (5) and the Medical School Learning Environment Survey (MSLES) (6).

The questionnaire was designed to assess students’ perceptions of several dimensions of the learning environment. It covered six domains: teaching quality, academic support, student well-being, social integration, curriculum relevance, and teacher-student interaction. The teaching quality domain addressed clarity of instruction, organization of teaching sessions, instructor preparedness, effectiveness of teaching methods, student engagement, and feedback. The academic support domain included academic guidance, faculty accessibility, mentoring, advising services, and learning resources. The student well-being domain assessed academic stress, work-life balance, emotional support, burnout, and overall educational satisfaction. The social integration domain covered students’ sense of belonging, peer relationships, teamwork, extracurricular participation, and collegial support. The curriculum relevance domain evaluated the perceived relevance of course content, integration of basic and clinical sciences, alignment with learning objectives, and preparation for future practice. The teacher-student interaction domain examined teacher approachability, respect for students, openness to discussion, fairness, and interaction during learning activities.

The questionnaire items were adapted from the DREEM and MSLES instruments to suit the local educational context; however, the instruments were not reproduced verbatim.

### Validity and pilot testing

- Content validation of the questionnaire was performed by a panel of medical education experts to ensure the clarity, relevance, and appropriateness of the items.
- A small pilot study was conducted among students who were not included in the final analysis to assess clarity and feasibility. Minor wording revisions were made accordingly.
- Internal consistency reliability was assessed using Cronbach’s alpha. A value of α ≥ 0.70 was considered acceptable; the observed overall reliability for the 53-item instrument was α = 0.91, with domain-level coefficients ranging from α = 0.78 to α = 0.88.

### Scoring system

Responses were measured on a 5-point Likert scale: 1 = strongly disagree, 2 = disagree, 3 = neutral, 4 = agree, and 5 = strongly agree. In line with commonly used DREEM item-level interpretation, mean scores of ≥3.5 were considered positive or strong areas, scores of 3.0–3.49 were considered moderate, and scores of <3.0 were considered areas requiring improvement. These thresholds were used to operationally classify strengths and weaknesses in the present study (17–19).

### Data collection procedure

The questionnaire was distributed electronically through university email and the learning management system. Participation was voluntary, and anonymity was assured.

Data were collected over a two-week period. Reminder messages were sent to improve the response rate. Completion of the questionnaire was considered to imply informed consent.

### Data analysis

Data were analyzed using SPSS version 28 (IBM Corp., Armonk, NY, United States). Data cleaning was performed to address missing values, coding errors, and inconsistencies.

The distribution of continuous variables was assessed before inferential analysis using visual inspection of histograms and Q-Q plots, together with the Shapiro–Wilk test. Skewness and kurtosis values were also examined to evaluate departures from normality. Variables that were approximately normally distributed were summarized using means and standard deviations, whereas non-normally distributed or highly dispersed Likert-scale items were additionally described using medians and interquartile ranges. Parametric tests were used because the main composite scores were approximately normally distributed and the sample size was sufficient for robust comparison. Descriptive statistics were used to summarize the data.

- Continuous variables are presented as means and standard deviations.
- Categorical variables are presented as frequencies and percentages.

Strengths and weaknesses evaluation

- Items with higher mean scores (≥ 3.5) were considered strengths.
- Items with lower mean scores (< 3.0) were considered weaknesses.

Inferential statistical analyses were performed as follows:

- Independent-samples *t*-test to compare gender differences.
- One-way ANOVA to assess differences across academic years.
- Tukey’s *post hoc* test to identify specific between-group differences.
- Pearson correlation analysis to examine relationships among domains.

Statistical significance was set at *p* ≤ 0.05.

### Ethical considerations

This study complied with ethical guidelines for research involving human participants. Ethical approval was granted by the Local Research Ethics Committee (LREC, Tabuk) of the University of Tabuk (Approval No. UT 3341702023, dated 18/12/2023). Participation was voluntary, and no personally identifiable information was collected. All data were stored on a password-protected, encrypted server accessible only to the core research team.

## Results

### Characteristics of participants and response rate

A total of 304 medical students from Years 1 to 6 participated in this study, yielding a response rate of 31.7% (304/960). Participants included students across all academic years (182 males and 122 females), providing representation from the broader student body.

### Overall perception of the learning environment

On the 5-point Likert scale, the overall mean score for perception of the learning environment (LE) was 3.52 (SD = 0.81), indicating a moderately positive perception. As shown in Table 1, the highest-rated items and their corresponding domains were:

**Table 1:** Overall learning environment assessment across all students (*n* = 304).

Top 5 strengths (highest mean scores)—item [domain]	Mean ± SD	Positive responses, *n* (%)	Top 5 weaknesses (lowest mean scores)—item [domain]	Mean ± SD; Median (IQR)	Positive responses, *n* (%)
The teachers are knowledgeable [Teaching quality]	4.1 ± 0.9	249 (82%)	I am too tired to enjoy the courses [Student well-being]	**2.36 ± 1.30; Median = 2.00, IQR = 1.00–4.00**	73 (24%)
I have good friends in this school [Social integration]	4.0 ± 1.0	237 (78%)	Cheating is a problem in the school [Social integration/school climate]	2.5 ± 1.3	85 (28%)
The teachers are patient with patients [Teacher–student interaction]	3.9 ± 1.0	228 (75%)	I am able to memorize all I need [Student well-being]	2.7 ± 1.3	94 (31%)
The teaching helps develop my competence [Curriculum relevance]	3.8 ± 1.0	219 (72%)	The teaching overemphasizes factual learning [Teaching quality]	2.8 ± 1.2	106 (35%)
My accommodation is pleasant [Student well-being]	3.8 ± 1.1	213 (70%)	I find my experience disappointing [Student well-being]	2.9 ± 1.3	116 (38%)
Overall composite score	3.5 ± 0.8	198 (65%)	–	–	–

Positive responses were defined as scores of 4 = agree or 5 = strongly agree on the 5-point Likert scale. The item “I am too tired to enjoy the courses” is negatively worded and was reverse-coded for interpretation. Median and interquartile range were reported for this item because of its relatively large standard deviation, indicating substantial variability in student responses. Bold values indicate the lowest score(s) highlighted to identify the weakest item requiring improvement.

Internal consistency was satisfactory: Cronbach’s alpha for the full questionnaire was α = 0.91, and domain-level alpha coefficients ranged from 0.78 to 0.88, supporting acceptable reliability of the composite and domain scores.

- “The teachers are knowledgeable”—Teaching quality (*M* = 4.12, SD = 0.88).
- “I have good friends in this school”—Social integration (*M* = 4.03, SD = 0.97).

The lowest-rated items and corresponding domains were as follows (Table 1):

- “I am too tired to enjoy the courses”—Student well-being (*M* = 2.31, SD = 1.22).
- “Cheating is a problem in the school”—Social integration/school climate (*M* = 2.47, SD = 1.31).

These findings suggest that teaching quality and social integration were perceived as major strengths, whereas student well-being and aspects of the broader school climate were the main areas of concern. The item “I am too tired to enjoy the courses” showed a low mean score with a relatively large standard deviation, indicating considerable variability in students’ responses. Therefore, this item should be interpreted cautiously, as fatigue may not have been experienced uniformly across all students or academic years.

### Variation by academic year

**Figure 1:**
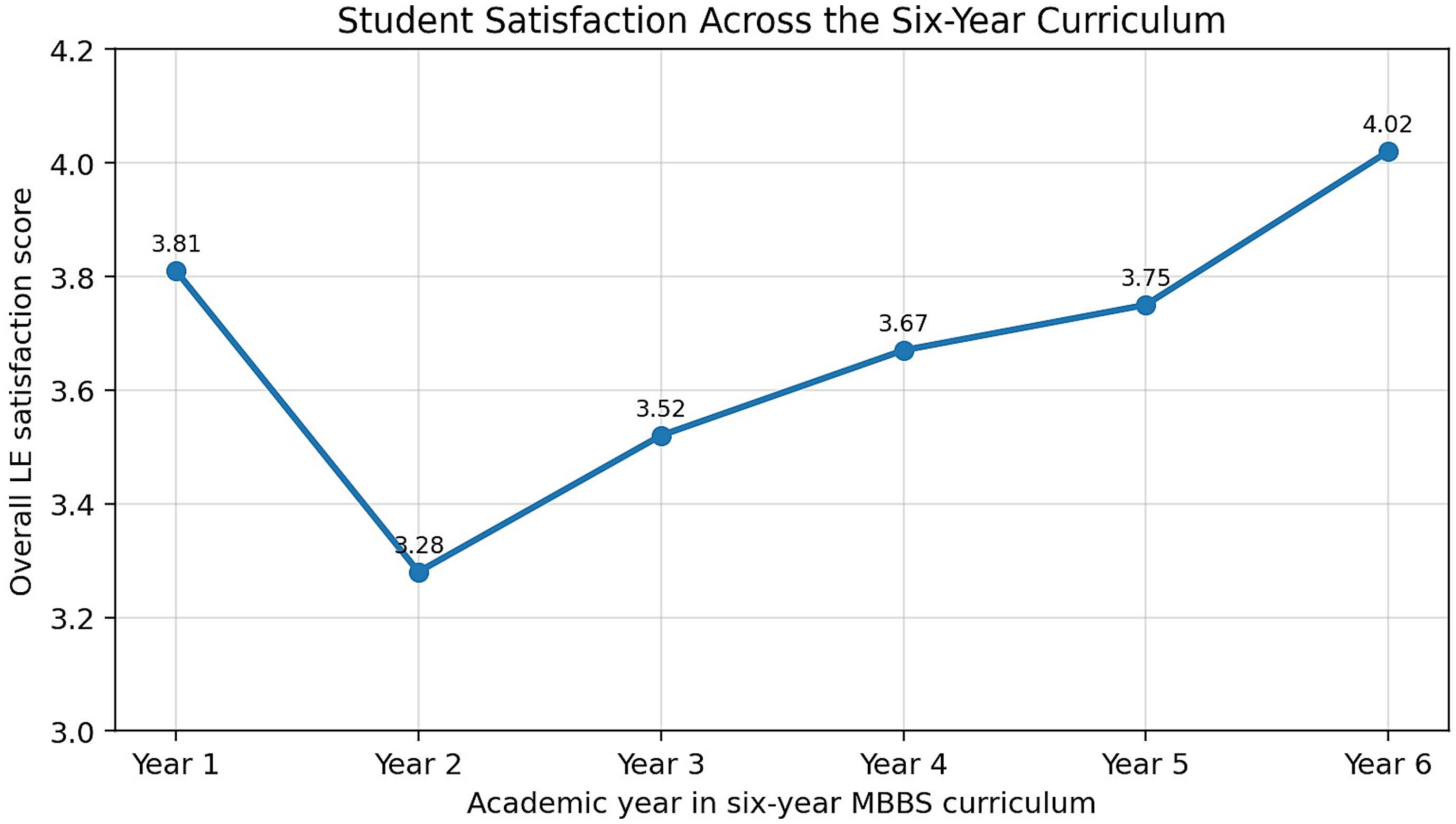
Student satisfaction across the 6-year MBBS curriculum. Mean overall learning-environment satisfaction is shown for Years 1 through 6 only, matching the 6-year MBBS curriculum. The lowest satisfaction occurred in Year 2, followed by progressive recovery during the clinical years.

A one-way ANOVA showed a statistically significant difference in LE perception across academic years (*F* = 9.87, *p* < 0.001). As shown in Table 2 and Figure 1, satisfaction followed a U-shaped pattern:

- Highest satisfaction in the first year (*M* = 3.81, SD = 0.72).
- The lowest satisfaction was in the second year (*M* = 3.28, SD = 0.89).
- Satisfaction increased progressively in the later years, with the highest scores observed in the sixth year (*M* = 4.02, SD = 0.61). *Post hoc* analysis confirmed significantly lower satisfaction among second-year students than among students in the other academic years (*p* < 0.01), suggesting that the second year may represent a particularly vulnerable stage in the curriculum.

**Table 2:** Learning environment dimensions by academic year.

Learning dimension	Description of items	1st year	2nd year	3rd year	Clinical years (4th–6th)	*F*-value	*p*-value
Teaching quality	Teacher knowledge, preparation, clarity, engagement	3.9 ± 0.7	3.4 ± 0.9	3.7 ± 0.8	4.0 ± 0.6	8.45	<0.001
Academic support	Academic guidance, support systems, learning atmosphere	3.6 ± 0.9	3.1 ± 1.1	3.3 ± 1.0	3.8 ± 0.8	6.32	<0.001
Student well-being	Fatigue, stress, motivation, emotional well-being	3.8 ± 0.8	3.2 ± 1.0	3.4 ± 0.9	3.9 ± 0.7	9.18	<0.001
Curriculum relevance	Professional preparation, skills, future relevance	3.7 ± 0.9	3.3 ± 1.0	3.6 ± 0.9	4.1 ± 0.7	7.89	<0.001
Social integration	Peer relationships, social life, support	4.0 ± 0.8	3.5 ± 1.1	3.7 ± 0.9	4.2 ± 0.6	5.96	<0.001
Teacher–student relations	Feedback, communication, interaction	3.5 ± 0.9	3.0 ± 1.1	3.2 ± 1.0	3.7 ± 0.8	5.43	<0.001
Overall satisfaction	Composite score of all items	3.8 ± 0.7	3.3 ± 0.9	3.5 ± 0.8	4.0 ± 0.6	10.23	<0.001

Clinical years (4th–6th) were grouped for comparison with preclinical years.

### Gender-related perception differences

**Figure 2:**
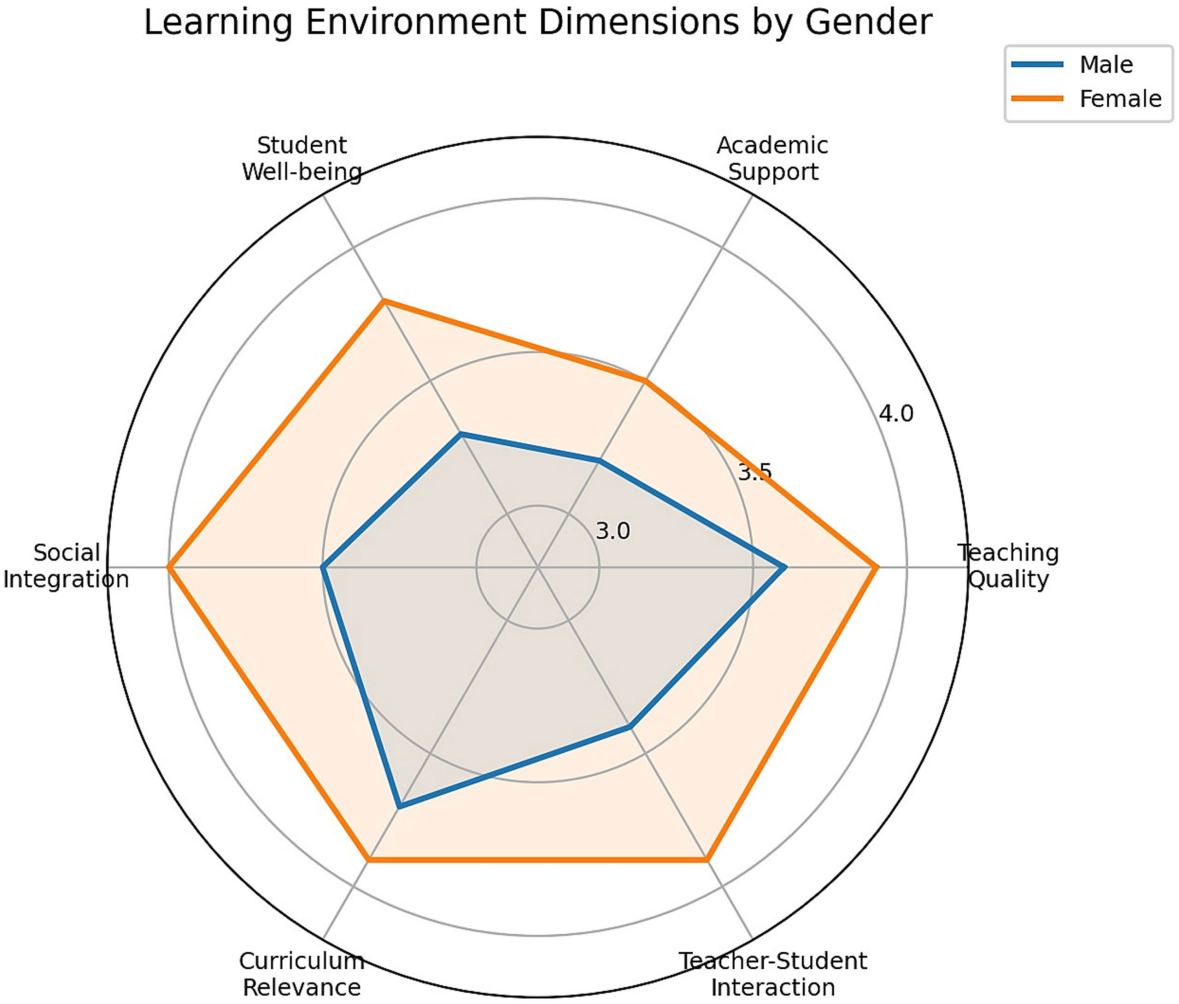
Radar chart comparing learning environment dimensions by gender. Female students reported higher mean scores than male students across the plotted domains. Domain labels correspond to the six-domain framework described in the Methods, with no duplicate domain labels.

Independent-samples *t*-tests revealed a statistically significant difference in overall LE perception by gender (Table 3 and Figure 2), with female students reporting significantly higher satisfaction than male students:

- Females: *M* = 3.79 (SD = 0.67).
- Males: *M* = 3.41 (SD = 0.89), *p* < 0.001, *d* = 0.50. The largest gender-related differences were observed in the following domains:
- Student well-being.
- Social integration.
- Teacher-student interaction.

**Table 3:** Gender differences in learning environment perceptions.

Key dimension	Male (*n* = 184)	Female (*n* = 120)	*t*-value	*p*-value	Cohen’s *d*
Teaching quality	3.30 ± 0.67	3.42 ± 0.53	−1.67	0.096	0.20
Academic support	3.05 ± 0.91	3.35 ± 0.75	−3.03	0.003	0.36
Student well-being	2.93 ± 0.75	3.14 ± 0.58	−2.65	0.008	0.31
Social integration	3.39 ± 0.59	3.53 ± 0.48	−2.12	0.035	0.25
Curriculum relevance	3.28 ± 0.87	3.47 ± 0.74	−1.88	0.061	0.22
Teacher–student interaction	3.27 ± 0.70	3.44 ± 0.62	−2.11	0.036	0.25
Overall satisfaction	3.21 ± 0.61	3.39 ± 0.47	−2.71	0.007	0.32

Values are mean ± SD. Independent-samples *t*-tests were used. Cohen’s d was calculated using the pooled standard deviation. Female scores were higher than male scores for all listed domains. The six prespecified domains are all included.

No statistically significant gender differences were observed in teaching quality and curriculum relevance.

### Relationship between learning environment constructs

Pearson correlation analysis showed strong positive relationships among the major domains of the learning environment (Table 4).

**Table 4:** Correlation matrix of learning environment domains and overall satisfaction.

Construct	1	2	3	4	5	6	7
1. Teaching quality	1.00						
2. Academic support	0.62**	1.00					
3. Student well-being	0.62**	0.65**	1.00				
4. Social integration	0.31**	0.30**	0.38**	1.00			
5. Curriculum relevance	0.72**	0.72**	0.66**	0.39**	1.00		
6. Teacher–student interaction	0.72**	0.61**	0.62**	0.39**	0.62**	1.00	
7. Overall satisfaction	0.86**	0.82**	0.82**	0.51**	0.87**	0.85**	1.00

Pearson correlation coefficients are reported. **p < 0.01. Constructs: 1 = Teaching Quality, 2 = Academic Support, 3 = Student Well-being, 4 = Social Integration, 5 = Curriculum Relevance, 6 = Teacher–Student Interaction, and 7 = Overall Satisfaction. The matrix includes all six learning-environment domains plus overall satisfaction.

The strongest relationship was observed between teaching quality and overall satisfaction (*r* = 0.85, *p* < 0.01). Academic support was also strongly associated with teaching quality (*r* = 0.72) and student well-being (*r* = 0.69).

There was also a substantial correlation between student well-being and social integration (*r* = 0.78). These findings suggest that improvements in teaching quality and academic support may have positive effects across multiple dimensions of the learning environment.

## Discussion

This study assessed medical students’ perceptions of the learning environment at Tabuk University and found an overall moderately positive educational climate, with significant differences across academic years and by gender. These findings suggest that the institutional environment is generally supportive, although several aspects remain in need of improvement. The overall mean LE score (3.52) is comparable to scores reported in other Saudi studies, which generally range from approximately 3.1 to 3.8 (15). This similarity suggests that the learning environment at Tabuk University broadly reflects national trends. However, relying solely on an overall score may obscure important subgroup differences.

The response rate of 31.7% should be considered when interpreting these findings. Although respondents represented all six academic years and both genders, non-response bias remains possible. Students with particularly positive or negative experiences may have been more motivated to participate, while students with high workload, fatigue, or disengagement may have been underrepresented. Consequently, the overall satisfaction score may either overestimate or underestimate the perceptions of the full student population. Future institutional surveys should combine repeated reminders, protected completion time, and comparison of respondents with non-respondents using administrative characteristics to better evaluate representativeness.

One of the key findings of this study was the U-shaped pattern of student satisfaction across the medical program, with the sharpest decline occurring in the second year and a gradual increase during the clinical years. This pattern resembles the “sophomore slump” described in the literature and may reflect increasing academic demands and the transition from introductory study to a more intensive, system-based preclinical curriculum (20, 21). The particularly low scores for student well-being in the second year indicate that this phase may represent a period of heightened academic and psychological stress. Previous studies have similarly shown that heavy workloads, high expectations, and reduced perceived support are associated with burnout and lower satisfaction (22).

The increase in satisfaction during the clinical years may be explained by greater patient contact, more hands-on learning experiences, and a clearer sense of professional identity. Clinical learning often enhances students’ motivation and engagement because it makes learning feel more meaningful and directly relevant to practice (23). These findings highlight the importance of strengthening the connection between preclinical teaching and clinical relevance in the early years of training.

Gender differences were also evident, with female students reporting higher satisfaction, particularly in the domains of social integration and well-being. This finding is consistent with several regional studies suggesting that female students may benefit from stronger social support networks and more favorable social environments (24). However, it differs from some international studies that reported no significant gender differences (25). This discrepancy suggests that cultural and institutional contexts may shape gendered experiences of the learning environment.

The identification of specific strengths and weaknesses is also useful for institutional improvement. High ratings for teacher knowledge and peer relationships indicate strong academic and social foundations within the school, which should be maintained. In contrast, low scores for fatigue, academic integrity concerns, and memorization highlight systemic challenges. Student fatigue, in particular, has been widely documented in medical education and is associated with poorer academic outcomes, reduced empathy, and a greater risk of burnout (26). These findings support further review of workload distribution, assessment practices, and curriculum structure to reduce unnecessary strain and promote meaningful learning.

Finally, the strong correlations among the learning environment domains underscore the interconnected nature of students’ educational experiences. The particularly strong association between teaching quality and overall satisfaction (*r* = 0.85) emphasizes the central importance of high-quality teaching. Likewise, the strong association between academic support and student well-being suggests that improving support systems may produce broad benefits across the learning environment. Taken together, these findings support a systems-based approach to educational improvement, in which targeted interventions in key areas may generate wider institutional benefits (27–31).

## Strengths and limitations

This study has several limitations. First, its cross-sectional design precludes causal inference. Second, although a census-based sampling strategy was used, the response rate was 31.7%, introducing potential non-response bias and limiting certainty about representativeness. Third, the findings are limited to a single institution and therefore may not be generalizable to other medical schools. Fourth, the 53-item questionnaire was derived from existing validated instruments (DREEM and MSLES), and although its content was reviewed for contextual relevance and internal consistency was acceptable, the instrument was not independently validated in this study population through exploratory or confirmatory factor analysis.

Fourth, although most findings were summarized using mean and standard deviation to allow comparison with previous DREEM-based studies, some Likert-scale items showed relatively large variability, particularly the item “I am too tired to enjoy the courses.” This variability suggests heterogeneous student experiences and indicates that future studies should report additional distributional statistics, such as median and interquartile range, for highly dispersed items.

## Conclusion

Tabuk University appears to provide a generally positive learning environment; however, satisfaction declined notably among second-year students, and significant gender differences were observed. Strengthening academic support during the preclinical years, promoting faculty development, and implementing student well-being initiatives may help improve the learning environment. Future longitudinal studies are warranted to assess change over time and evaluate the impact of targeted interventions.

## Recommendations

To promote a supportive institution-wide climate, the university should develop targeted wellness and academic support initiatives for second-year students; review preclinical workload and assessment practices to reduce fatigue and excessive rote learning; strengthen faculty development in inclusive pedagogy and student engagement; examine factors contributing to the positive social integration reported by female students; reinforce positive social and academic integration across the institution; and establish an annual LE assessment cycle to monitor trends and evaluate the impact of interventions.

## Data Availability

The original contributions presented in the study are included in the article/supplementary material, further inquiries can be directed to the corresponding author.
